# The deregulated microRNAome contributes to the cellular response to aneuploidy

**DOI:** 10.1186/s12864-018-4556-6

**Published:** 2018-03-14

**Authors:** Milena Dürrbaum, Christine Kruse, K. Julia Nieken, Bianca Habermann, Zuzana Storchová

**Affiliations:** 1 Max Planck Institute of Biochemistry, Am Klopferspitz 18, 82152 Martinsried, Germany; 2 Center for Integrated Protein Sciences Munich, Ludwig-Maximilians-Universität München, Butenandtstr. 5, 81377 Munich, Germany; 3 Computational Biology Group, Developmental Biology Institute of Marseille (IBDM) UMR 7288, CNRS, Aix Marseille Université, 13288 Marseille, France; 4 Department of Molecular Genetics, TU Kaiserslautern, Paul Ehrlich Strasse 24, 67663 Kaiserslautern, Germany

**Keywords:** Aneuploidy, Cancer, miRNA, miR-10a-5p, Trisomy

## Abstract

**Background:**

Aneuploidy, or abnormal chromosome numbers, severely alters cell physiology and is widespread in cancers and other pathologies. Using model cell lines engineered to carry one or more extra chromosomes, it has been demonstrated that aneuploidy *per se* impairs proliferation, leads to proteotoxic as well as replication stress and triggers conserved transcriptome and proteome changes.

**Results:**

In this study, we analysed for the first time miRNAs and demonstrate that their expression is altered in response to chromosome gain. The miRNA deregulation is independent of the identity of the extra chromosome and specific to individual cell lines. By cross-omics analysis we demonstrate that although the deregulated miRNAs differ among individual aneuploid cell lines, their known targets are predominantly associated with cell development, growth and proliferation, pathways known to be inhibited in response to chromosome gain. Indeed, we show that up to 72% of these targets are downregulated and the associated miRNAs are overexpressed in aneuploid cells, suggesting that the miRNA changes contribute to the global transcription changes triggered by aneuploidy. We identified hsa-miR-10a-5p to be overexpressed in majority of aneuploid cells. Hsa-miR-10a-5p enhances translation of a subset of mRNAs that contain so called 5’TOP motif and we show that its upregulation in aneuploids provides resistance to starvation-induced shut down of ribosomal protein translation.

**Conclusions:**

Our work suggests that the changes of the microRNAome contribute on one hand to the adverse effects of aneuploidy on cell physiology, and on the other hand to the adaptation to aneuploidy by supporting translation under adverse conditions.

**Electronic supplementary material:**

The online version of this article (10.1186/s12864-018-4556-6) contains supplementary material, which is available to authorized users.

## Background

A balanced karyotype is essential for cell viability and therefore aneuploidy, characterized by unbalanced changes in chromosome numbers and sub-chromosomal structural variations, has often profound detrimental consequences for cell physiology. In humans, aneuploidy is the major cause of spontaneous abortions and the few trisomies compatible with survival result in severe developmental defects [1]. Aneuploidy in somatic cells is frequently associated with cancer, as 70% of haematopoietic and 90% of solid cancers show an abnormal karyotype [2, 3]. Recently developed aneuploid model systems in several different species have accelerated research on the effects of aneuploidy *per se*. The physiological changes in response to an unbalanced karyotype are multifold, including impaired proliferation, replication stress and proteotoxic stress that is characterized by changes in protein stoichiometry, reduced protein folding capacity and by activation of autophagy [4–13]. The physiological response to aneuploidy goes hand in hand with a transcriptional response that is manifested in a conserved pathway deregulation [14, 15]. What triggers these changes in gene expression and how exactly this specific response is modulated has not been clarified so far.

Given that the triggers of the transcriptional deregulations remain unclear, we asked whether microRNA (miRNA) regulation is involved in the response to aneuploidy. MiRNAs are small non-coding RNA molecules that posttranscriptionally modulate gene expression of over 60% of protein coding genes [16]. The regulatory function of miRNAs is mediated by their binding to the target 3’untranslated region (UTR) via partially complementary sequences, thereby inducing translational repression and/or mRNA decay. Alternatively, some miRNAs bind to sites within the coding region or the 5’UTR [17, 18]. This binding can potentially stabilize the mRNA or enhance its translation [19]. One mRNA can be affected by multiple miRNAs that cooperatively translationally repress or degrade the target mRNA or compete for target regulations [20]. In turn, single miRNA can repress hundreds of mRNAs and lead to large-scale transcriptome changes that for example play a key role in stem cell differentiation [21]. Moreover, the complex miRNA-target network allows to fine-tune diverse cellular processes by modulating the amount of transcripts translated particularly upon stress conditions or changes in the environment [22, 23].

In the disease context such as in cancer, alterations of miRNA expression are common and specific miRNA deregulation profiles are sufficient to distinguish cancerous from non-cancerous tissues and to predict invasiveness and aggressiveness of various cancers [24–26]. The widespread miRNA deregulation in cancers has been attributed to genomic copy number changes. For instance, a gain of chromosome 1q relates to miRNA expression changes in cervical cancer [27] and downregulation of let-7 family was associated with copy number changes in medulloblastoma, breast and ovarian cancer [28]. In some cancers, deregulated miRNAs promote genomic and chromosomal instability by targeting the mitotic checkpoint or DNA damage repair components [29, 30]. Yet, beyond these few examples, we lack a deeper understanding of how aneuploidy in cancer and miRNAs expression changes are linked. Moreover, the relation of miRNA and aneuploidy *per se* has not been studied so far.

**Fig. 1:**
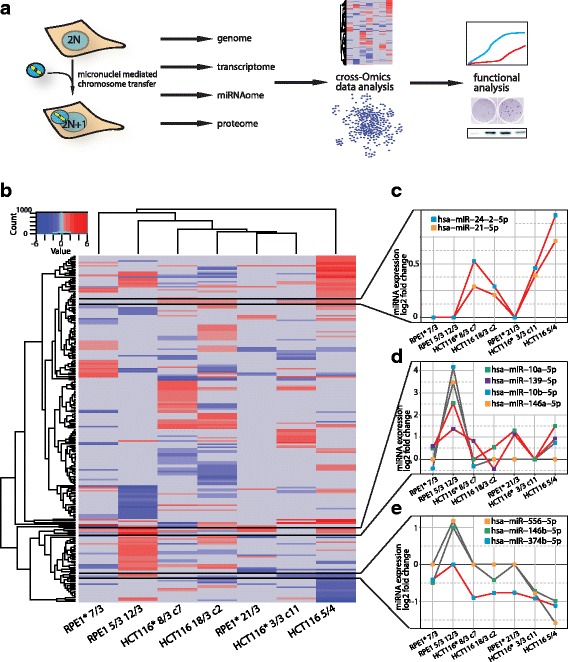
miRNAome in aneuploid model cell lines. **a** Schematic summary of the work flow. **b** Heat map of significantly altered miRNAs in seven different aneuploid cell lines (adjusted *p*-value <=0.05). Blue indicates downregulation, red upregulation. Row and column dendrograms show Euclidean distance between miRNA expression profiles and cell lines, respectively. Commonly deregulated miRNA clusters are highlighted by black lines. **c** miRNA expression profile of miRNA cluster upregulated in all HCT116-derived aneuploid cell lines. **d** miRNA expression profile of miRNA cluster containing hsa-miR-10a-5p and hsa-miR-139-5p that are upregulated in 5 out of 7 sequenced aneuploid cell lines (red line). Grey lines indicate miRNAs in the same cluster that are not commonly deregulated. **e** miRNA expression profile of miRNA cluster containing hsa-miR-374b-5p that is down regulated in 6 out of 7 sequenced aneuploid cell lines (red line). Grey lines indicate miRNAs in the same cluster that are not commonly deregulated. Cell lines were ordered according to Euclidean distance. Asterisks indicate cell lines with H2B-GFP

We asked whether aneuploidy affects miRNA abundance and analysed the phenotypic consequences of miRNA changes in aneuploid cells (Fig. 1a). To this end, we used a series of human trisomic and tetrasomic cell lines that very previously constructed in our laboratory by micronuclei-mediated chromosome transfer into diploid, chromosomally stable human cell lines HCT116 and RPE1 ([12], for further details see Methods). By transferring the individual chromosome, we obtained homogeneous population with a defined aberrant karyotype. Our model cell lines therefore allow study the general consequences of chromosome gain by direct comparison of different trisomies and tetrasomies to their isogenic parental cells. Using this model system, we found that chromosome gains strongly deregulate the expression of more than 25% of miRNAs, although only a few individual miRNAs were commonly altered among different aneuploidies. Most of the identified miRNA deregulations negatively affect cell growth and proliferation, thus suggesting that miRNAs suppress proliferation of aneuploid cells. We identified miRNA hsa-miR-10a-5p to be overexpressed in majority of analysed cell lines. This miRNA acts through both the canonical 3’UTR targeting as well as via the 5’UTR of target mRNAs. We show that the alternative 5’UTR targeting provides the aneuploid cells with resistance to starvation by ensuring sustained translation of ribosomal mRNAs upon serum withdrawal. Our analysis of the global miRNAome changes in response to chromosome gain reveals a complex interplay of the gene expression regulatory mechanisms that shape the global transcriptome and proteome dynamics in aneuploid cells.

## Results

### Deregulation of miRNAome in human aneuploid model cell lines

To determine the effects of chromosome gain on miRNA expression in human cells, we used a series of cells derived from HCT116 and RPE1 cell lines that contain one or more extra copies of different chromosomes ([5, 9, 12], Fig. 1a, see Methods for more details). Following cell lines were subjected to small RNA sequencing: four HCT116- derived cell lines that gained chromosome 3, 8, 5 or 18, respectively, the parental HCT116 cell line, three RPE1- derived cell lines trisomic for chromosome 7, 21, cell line trisomic for both chromosome 5 and 12 and the parental RPE1 cell line. The miRBase repository [31], where 1881 mature human miRNAs are listed to date, was used as the miRNA data source for mapping. Mapping of the raw sequences to the human genome and subsequent identification of miRNAs with mirdeep2 [32] resulted in at least 554 and up to 719 identified mature miRNAs in the sequenced cell lines (Table 1).

**Table 1:** Detected mature miRNAs

	HCT116			HCT116*v (with H2B-GFP)			RPE1		RPE1* (with H2B-GFP)		
	control	5/4	18/3 c2	control	3/3 c11	8/3 c7	control	5/3 12/3	control	7/3	21/3
mature miRNAs in mirBase	1881										
miRNAs detected by mirdeep2	719	554	592	747	572	562	650	628	647	663	646
miRNAs detected in every sample	626	486	510	647	485	490	566	564	562	575	564

The asterisk in the cell line name marks the presence of H2B-GFP

While the numbers of identified miRNAs were similar for all sequenced aneuploid cell lines, the extent of miRNA deregulation differed. We analyzed the differential miRNA expression in aneuploid cell lines in comparison to their diploid counterparts by applying DESeq2 for normalization and statistical testing [33]. Since miRNAs with very low expression levels are characterized by low read counts and an inherent large variability on the logarithmic scale, these miRNAs were pre-filtered [33]. This filtering for miRNAs with a read count greater than 10 reduced the number of identified miRNAs to 231–293 (Table 2). Using this dataset, we then determined the significantly deregulated miRNAs as miRNAs with the aneuploidy/diploid log2 fold change +/− 0.6 at adjusted *p*-value < 0.05. We identified from 23 (in RPE1* 21/3) to 74 (in RPE1 5/3 12/3 and HCT116 5/4) significantly deregulated miRNAs (Table 2). The percentage of deregulated miRNAs ranged from 10% (in RPE1* 21/3) up to 31.2% (in RPE1 5/3 12/3). There was only a weak correlation between the percentages of deregulated miRNAs with the amount of extra DNA (Additional file 1: Figure S1). Thus, gain of even a single chromosome deregulates up to one third of cellular miRNAs.

**Table 2:** Number of deregulated miRNAs and mRNAs in aneuploid cell lines

	HCT116				RPE1		
	5/4	18/3 c2	^*^3/3 c11	^*^8/3 c7	5/3 12/3	^*^7/3	^*^21/3
miRNAs with a mean count ≥ 10	249	280	279	293	237	247	231
Significantly deregulated miRNAs log2 Fold Change 0.6–0.6 (padj. < 0.05)	74	57	27	44	74	33	23
Percentage of significantly deregulated miRNAs	29.7	20.4	9.7	15.0	31.2	13.4	10.0
mRNAs with a mean count ≥ 10	13,887	n.a.	n.a.	13,873	13,278	13,101	13,101
Significantly deregulated mRNAs log2 Fold Change 0.6–0.6 (padj. < 0.05)	2885	n.a.	n.a.	410	2210	434	434
Percentage of significantly deregulated mRNAs	20.8	n.a.	n.a.	3.0	16.6	n.a.	3.3

Cell lines with ^*^ contain H2B-GFP

### Cell line specific and general miRNA deregulation in response to aneuploidy

Comparison of the significantly altered miRNAs in aneuploid cell lines revealed a pattern of miRNA expression changes largely distinct for each specific cell line (Fig. 1b). The *Euclidean* distance clustering was not defined by the parental cell type, but rather by distinct and cell line specific miRNA expression clusters individual for each aneuploid cell line. HCT116 5/4 showed the most prominent clusters of down- and upregulated miRNAs and appeared as an outlier in the *Euclidean* distance cluster. The upregulated miRNA cluster in HCT116 5/4 partially overlapped with the upregulated miRNAs in RPE1 5/3 12/3; among them most prominently the miRNA family miR-192/215, the nearby located miR-194 and miRNAs hsa-miR-29b and hsa-miR-29c, which are encoded in a cluster on chromosome 1. Only a few similarities were found among different cell lines. For instance, hsa-miR-21-5p and hsa-miR-24-2-5p showed similar upregulation in all HCT116-derived cell lines (Fig. 1c). Moreover, five out of seven sequenced aneuploid cell lines shared the upregulation of hsa-miR-139-5p and hsa-miR-10a-5p (Fig. 1d). The latter upregulation did not affect the entire miRNA family and seems to be specific for the individual miRNA: while hsa-miR-10a-5p was upregulated in five out of seven of the analyzed cell lines, the family member hsa-miR-10b-5p was upregulated only in RPE1 5/3 12/3. Only a few globally downregulated miRNAs were identified, such as hsa-miR-374b-5p that was downregulated in all cell lines except RPE1 5/3 12/3 (Fig. 1e).

**Fig. 2:**
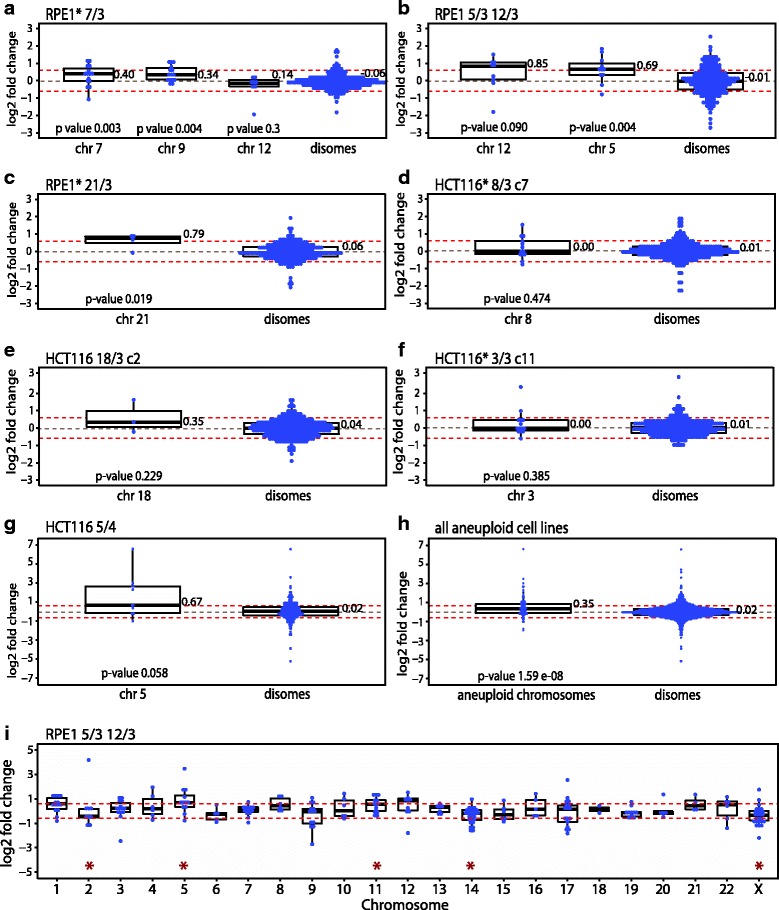
Expression of microRNAs encoded on the aneuploid chromosomes compared to expression of microRNAs encoded on disomic chromosomes. Comparison of the expression changes of miRNAs from the aneuploid chromosome and for the miRNAs of the remaining disomic chromosomes in individual cell lines. **a** RPE1* 7/3 (note that this cell line spontaneously gained additional copy of chromosome 9 and 12). **b** RPE1* 21/3, **c** RPE1 5/3 12/3, **d** HCT116 5/4, **e** HCT116 18/3 clone 2, **f** HCT116* 3/3 clone 11, **g** HCT116* 8/3 clone 7, and **h** Expression fold changes of miRNAs of all aneuploid chromosomes from all analysed cell lines compared to all disomic miRNAs from all cell lines. **i** Representative blot of the expression changes of miRNAs on each individual chromosome for RPE1 12/3 5/3. Blots for the remaining cell lines are in in Additional file 1: Figure S2. *For all blots*: Each blue dot represents a microRNA, the log2 fold change expression is always normalized to the expression levels of the corresponding parental cell line. Boxplot present 75%, 50% and 25% quantile; the median values are shown. Red dotted lines marked log2 of 2-fold change (0.67). Significance was tested with Mann-Whitney-Wilcoxon test (marked with red asterisk in panel **i**). Cell lines with * contain H2B-GFP

The presence of extra chromosomes in cells generally leads to an elevated expression of the genes that are located on the supernumerary chromosomes; thus the abundance of transcripts scales with the gene copy numbers [6, 12–15, 34, 35]. To determine whether this holds also true for miRNA expression, we ordered the miRNAs according to their chromosome location. We then compared the distribution of the miRNA expression from the trisomic/tetrasomic chromosomes to miRNA expression from the disomes and tested whether the elevated expression occurred just by chance. The chromosome-specific miRNA expression was significantly higher than the disomic miRNA expression only for RPE1* 7/3, RPE1* 21/3 and for the miRNAs encoded on chromosome 5 in RPE1 5/3 12/3 (Fig. 2a–c). In all other aneuploid cell lines, the miRNA expression distribution did not increase significantly with the number of chromosome copies (Fig. 2c–g, Additional file 2: Table S1). In addition, the median miRNA expression differed from the expected levels even for some disomic chromosomes in several cell lines (Additional file 1: Figure S2A). For instance, the median expression of miRNAs encoded on chromosome 11 and 17 were significantly increased in HCT116 5/4 despite their disomic status that was confirmed by DNA sequencing. Taken together, gain of even a single chromosome leads to strong deregulation of up to 30% of miRNAs. The largely increased abundance of the deregulated miRNAs cannot be explained by the chromosome copy number changes.

### Effects of the deregulated miRNAome on transcriptome

We asked to what extent the mRNA expression changes in response to aneuploidy can be explained by miRNA deregulation. The transcriptome of HCT116 5/4, HCT116* 8/3 c7, RPE1 5/3 12/3 and RPE1* 21/3 was sequenced. The percentage of deregulated mRNAs (log2 fold change +/− 0.6, adjusted *p*-value < 0.05) highly differed among the cell lines, with 3.3% in RPE1* 21/3, 16.6% in RPE1 5/3 12/3, 3.0% in HCT116* 8/3 c7 and 20.8% in HCT116 5/4 (Table 2). To model the miRNA-target network, we retrieved reported miRNA targets from miRTarBase (v6.1) for all deregulated miRNAs of each cell line [36]. We then filtered these miRNA targets for those that were altered in response to aneuploidy. Although in principle each target can be affected by several miRNAs, we simplified the complex miRNA-mRNA relation and filtered for pairs of individual deregulated mRNA targets that were inversely expressed relative to the individual corresponding miRNAs. This analysis revealed that 38% of mRNAs that are deregulated in HCT116 5/4 are potentially targeted by the deregulated miRNAs, as judged by their inverse expression, 24% in RPE1* 21/3, 27% in RPE1 5/3 12/3 and only 4.8% HCT116* 8/3 c7 (Table 3). Thus, under these approximations, only a minor fraction of the mRNA changes in response to aneuploidy might be directly influenced by miRNA regulation.

**Table 3:** Numbers of targets of deregulated miRNAs (DEmiRNAs)

Cell line	HCT116 5/4	RPE1 5/3 12/3	RPE1* 21/3	HCT116 8/3 c7
Deregulated mRNAs	3342	2484	788	250
Deregulated miRNAs (DEmiRNA)	74	74	23	44
ALL TARBASE TARGETS				
Number of targets of DEmiRNAs				
Total miRNA-target interactions	9111	6487	6036	965
Unique interactions	6644	4605	5078	877
% of those deregulated on mRNA level	38.3% (1280)	24.3% (604)	27.2% (214)	4.8% (12)
Targets of DEmiRNAs with inverse expression:				
Total interactions	904	406	142	7
Unique interactions	729	345	127	7
TARBASE TARGETS WITH STRONG EVIDENCE				
Number of targets of DEmiRNAs				
Total miRNA-target interactions	729	546	496	213
Unique interactions	520	417	431	193
% of those deregulated on mRNA level	4.0% (133)	2.7% (68)	4.0% (20)	2.8% (6)
Targets of DEmiRNAs with inverse expression:				
Total interactions	154	54	20	3
Unique interactions	93	40	13	3

The asterisk in the cell line name marks the presence of H2B-GFP

### Deregulated miRNAs negatively affect cell cycle associated pathways

**Fig. 3:**
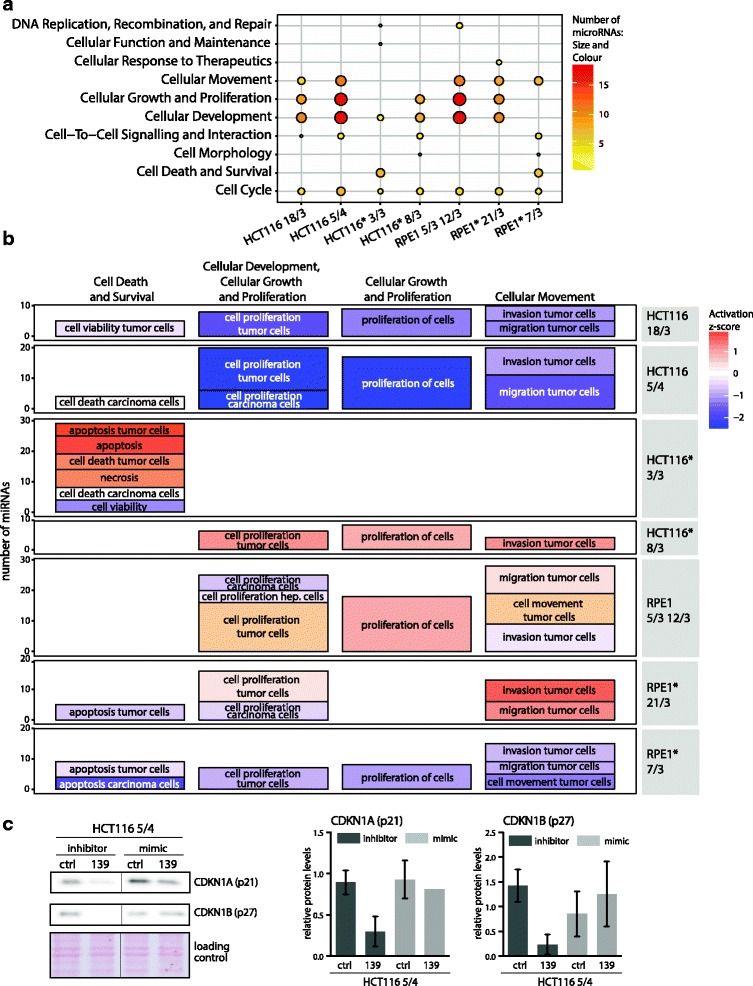
microRNAome of aneuploid cells affects cellular development, growth and proliferation. **a** Top ten cellular and molecular functions affected by the deregulated microRNAs in individual aneuploid cell lines (in alphabetical order bottom-to-top). The dot size and colour outlines the individual data and indicates the number of microRNAs that are associated with a specific cellular function. **b** Subfunctional annotation terms for the four cellular and molecular functions with the most miRNAs involved (columns). Each graph row contains subfunctional terms for one aneuploid cell line. The size of the boxes indicate the number of microRNAs associated (y-axis); the colour indicates the predicted activation z-score. Asterisks indicate cell lines with H2B-GFP. Hep. cells abbreviates hepatoma cell lines. **c** Immunoblotting of negative cell cycle regulators p21 and p27 upon transfection with inhibitors and mimics of miRNA139-5p. Left: Example of the immunoblotting, Ponceau S staining was used as a loading control. Right: Quantification based on four independent experiments, mean and standard deviation are shown

To determine which cellular functions are affected by the changes in the miRNAome in aneuploid cells, we performed functional analysis based on the manually curated knowledge base of Ingenuity Pathway Analysis [37], where the miRNA annotations are derived from their published functional roles, independently of their validated and predicted targets. Pathway “Cell Cycle” was affected in all aneuploid cell lines and additional four molecular and cellular functions affected by the deregulated miRNAs were shared in at least five out of seven aneuploid cell lines (Fig. 3a). The miRNAome of HCT116* 3/3 showed less functional associations and a low number of involved miRNAs, although pathways “Cellular Development” and “Cell Cycle” were also affected (Fig. 3a).

We employed IPA to infer an activation state of the biological functions as an activation z-score. The IPA activation z-score is a quantitative measure of the predicted effect of the deregulated miRNAome on a biological function [38]. It is calculated based on the direction of miRNA deregulation and the literature-derived positive or negative effects of these miRNAs on a biological function. The available miRNA dataset allowed to retrieve the z-score for the sub-functional annotation terms defined by IPA within the categories “Cell Death and Survival”, “Cellular Development, Cellular Growth and Proliferation”, “Cellular Growth and Proliferation” and “Cellular Movement” (Fig. 3b). The effect of the deregulated miRNAome was mostly negative, as the sub-functional annotation terms within the four parent categories were predicted to be inhibited or deactivated in HCT116 18/3, HCT116 5/4 and RPE1* 7/3. The activation z-score of HCT116* 3/3 miRNA deregulation predicted the changes for the category “Cell Death and Survival”, which is in concordance with the identity of the five top molecular and cellular functions (Fig. 3). Therein, “apoptosis” was predicted to be activated with a z-score of 1.8 and 2 and “cell death” as well as “necrosis” with a z-score of 1.5 and 1.4, respectively. Thus, the negative impact of miRNA deregulation might be executed in HCT116* 3/3 rather via upregulation of “Cell Death” pathways than by downregulation of “Cellular Growth and Proliferation” pathways.

To examine the effect of identified miRNAs on cell cycle, we transfected mimics and inhibitors of the only two miRNA candidates that were upregulated in at least five out of seven analysed aneuploid cell lines: miR-10a-5p and miR-139-5p (Fig. 1b-d). Whereas transfections of the hsa-miRNA-10a-5p mimics and inhibitors had only a negligible effect on proliferation and expression of cell cycle markers (data not shown), we found that inhibition of miRNA 139-5p indeed decreased the levels of negative cell cycle regulators CDKN1A (p21) and CDKN1B (p27) (Fig. 3c). MiRNA-139-5p is located on chromosome 11 at 11q13.4 and its expression is downregulated in a variety of cancers including hepatocellular carcinoma, gastric cancer, breast cancer and glioblastoma (e.g [39]). In summary, although the deregulated miRNAs differed in individual cells lines, we found a striking overlap in their predicted, largely negative impact on cellular growth and proliferation.

### Integration of miRNA, RNA and protein expression data confirms the negative effect on cellular development, cellular growth and proliferation

**Fig. 4:**
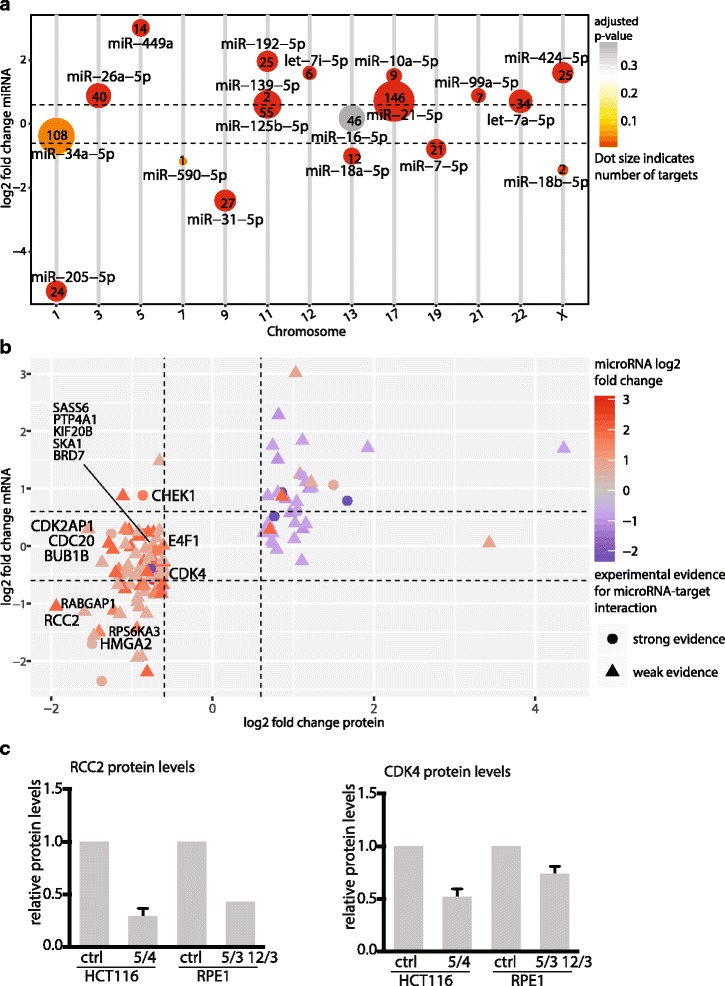
microRNA target expression infers functional role of microRNAs. **a** Number of targets with a strong evidence for each deregulated microRNA annotated in the “Cellular Development, Cellular Growth and Proliferation” category in HCT116 5/4. The size indicates the number of targets, the colour shows the significance of microRNA deregulation. The microRNAs are presented according to their chromosomal location. **b** Target genes within the “Cellular Development, Cellular Growth and Proliferation” category and their mRNA and protein expression. Selected gene labels indicate targets with inverse miRNA-target expression that are associated with cell cycle processes. Shape indicates the type of experimental evidence for a microRNA-target interaction. The colour indicates log2 fold change microRNA expression. **c** Quantification of the protein levels of miRNA targets RCC2 and CDK4 in aneuploid and parental control cell lines. The quantification is based on four independent immunoblotting experiments, mean and standard deviation are shown

To illuminate how the miRNAs execute their negative impact on cellular development, growth and proliferation in aneuploids, we analyzed their targets. For this purpose we exploited the fact that complete miRNAome, transcriptome and proteome data were available for three cell lines (HCT116 5/4, RPE1 5/3 12/3 and RPE1* 21/3, this work and [12]). In total, we identified 40 aneuploidy-responsive miRNAs to be associated with the cellular development, growth and proliferation category (Additional file 1: Figure S3A). Comparison with experimentally validated miRNA-target interactions revealed 604 interactions with 325 unique targets for HCT116 5/4 (Fig. 4a), 238 in RPE1 5/3 12/3 and 208 in RPE1* 21/3 (Additional file 1: Figure S3B, C). Chromosome alignment of the miRNAs revealed no bias for the superfluous chromosome in any of the cell lines.

To infer the effect of miRNA regulation on experimentally validated targets, we matched the transcriptome and proteome data to the targets of miRNAs within the cellular development, growth and proliferation category. Targets were filtered for their expression changes above or below a threshold ratio of +/− 0.6 (log2 fold change). Only experimentally validated targets with a potential miRNA relation (that means with inverse expression to the interacting miRNA) were considered for the annotation enrichment analysis. Strikingly, the protein abundance of 72% of the filtered targets was downregulated in HCT116 5/4, many of them being known cell cycle and proliferation- associated proteins (Fig. 4b). This observation holds also true for RPE1 5/3 12/3 and RPE1* 21/3 (Additional file 1: Figure S3D, E). Thus, the target gene expression analysis substantiates the predicted activation state of the molecular function and suggests a global negative effect of the miRNA changes on cell cycle and proliferation in response to aneuploidy.

Additionally, we performed functional annotation clustering of the miRNA targets with the DAVID functional annotation tool. This analysis confirmed the cell cycle functions as the most affected cluster (Additional file 3: Table S2). Among the deregulated targets in HCT116 5/4 we found key players of proliferation, such as the mitotic checkpoint serine/threonine kinases (BUB1), cell division cycling 20 (CDC20), cyclin-dependent kinase 4 (CDK4), checkpoint kinase 1 (CHEK1), high motility group A2 (HMGA2) and regulator of chromosome condensation (RCC2) (Fig. 4b). Cell cycle associated factors were also targeted in RPE1 5/3 12/3 and RPE1* 21/3, such as the minichromosome maintenance protein complex members (MCM2, 3 and 6) and Cyclin E1 (Additional file 1: Figure S3D, E). Immunoblotting of some of the targets confirmed their downregulation in response to aneuploidy (Fig. 4c). Taken together, deregulated miRNAome in aneuploid cells negatively affects expression of cell cycle factors and thereby may contribute to the proliferation defect observed in aneuploid model cell lines.

### Hsa-miR-10a-5p activity is increased in human aneuploid model cell lines

**Fig. 5:**
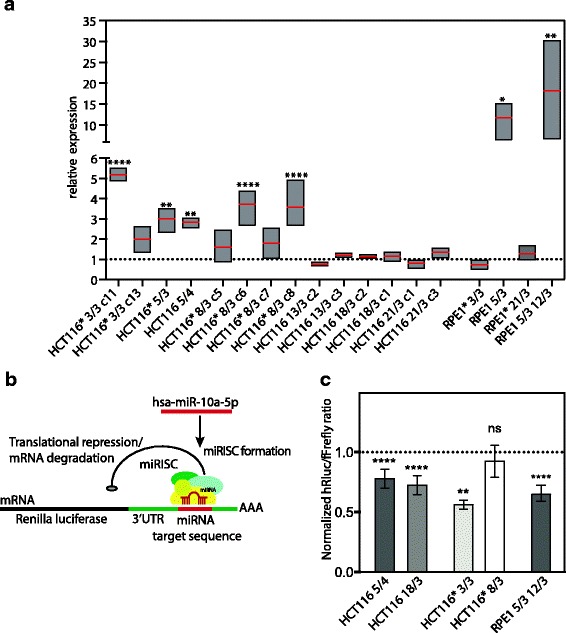
hsa-miR-10a-5p levels and activity are increased in aneuploid cel lines. **a** hsa-miR-10a-5p expression analysed by quantitative real-time PCR in 18 different aneuploid model cell lines. Boxplots present minimal, maximal and the mean value. Relative expression levels normalized to corresponding parental cell line is shown. **b** Luciferase reporter construct to determine hsa-miR-10a-5p endogenous gene repression activity. Hsa-miR-10a-5p targets its binding site in a synthetic 3’UTR of the Renilla luciferase mRNA resulting in translational repression and/or degradation of Renilla luciferase mRNA. Renilla luciferase signal is normalized to the internal Firefly luciferase control (see Methods). **c** Endogenous hsa-miR-10a-5p activity determined by psiCheck2-10a luciferase reporter assay with hsa-miR-10a-5p binding site in synthetic 3’UTR. HCT116-derived and RPE1-derived cell lines were transfected with psiCheck2-10a luciferase reporter construct. Luciferase reporter assay was conducted 48 h post transfection. Data represent the normalized mean values +/− SEM from at least three independent experiments, each performed in triplicates. One-way ANOVA, multiple comparison correction with Dunnett test. ns = not significant, *P < 0.0332, **P < 0.0021, ***P < 0.0002, ****P < 0.0001. Asterisks indicate the cell lines with H2B-GFP

While the majority of deregulated miRNAs were not shared among aneuploid cell lines, the miRNA hsa-miR-10a-5p was significantly upregulated in five out of seven aneuploid cell lines. Quantitative real-time PCR of 18 different aneuploid model cell lines (for details see Material and Methods) revealed that the mean expression of hsa-miR-10a-5p was significantly increased above the levels of the parental cell line in 7 out of 18 cell lines and elevated in additional 8 cell lines (Fig. 5a). Interestingly, the trisomies of chromosome 13, 18 and 21 that are compatible with survival in a whole organismal context (Patau, Edwards and Down syndrome, respectively) showed no or only negligible increase of hsa-miR-10a-5p expression. The increased expression of hsa-miR-10a-5p might be a secondary effect of the expression of HOXB3, in whose locus the miRNA sequence is located. Comparison of the hsa-miR-10a-5p and HOXB3 expression levels in four cell lines revealed that both miR-10a-5p and HOXB3 were significantly upregulated only in two cell lines, HCT116 5/4 and RPE1* 21/3, while their levels were independent in the other cell lines (Additional file 1: Figure S4). Thus, the upregulation of hsa-miR-10a-5p cannot be generally explained by increased expression of the host gene HOXB3.

To determine whether hsa-miR-10a-5p overexpression affects gene expression in aneuploid cells, we used a luciferase reporter assay to evaluate its translational repression efficiency (Ørom et al., 2008). The luciferase reporter consists of a miR-10a binding site in a synthetic 3’untranslated region (UTR) of the Renilla luciferase gene (Fig. 5b). The reporter additionally contains a Firefly luciferase gene that allows normalization for transfection efficiency. Indeed, the normalized luciferase signal was significantly lower in four out of five tested aneuploid cell lines (Fig. 5c). No changes in luciferase signal were detected in the HCT116* 8/3 c7 cell line; this is likely due to a relatively minor increase in the hsa-miR-10a-5p expression in this cell line (see Fig. 5a).

The target database lists 298 unique target genes for hsa-miR-10a-5p with only nine previously validated targets. Only six of the targets were downregulated on both transcriptome and proteome level in HCT116 5/4 (Additional file 1: Figure S5A). Only two of the 270 hsa-miR-10a targets were downregulated on transcriptome and proteome in both RPE1 5/3 12/3 and RPE1* 21/3 (Additional file 1: Figure S5B,C). This is in contrast with the other identified deregulated miRNAs, where majority of the targets showed inversed expression levels and suggests that the hsa-miR-10a-5p function in aneuploids might be independent of the 3’UTR target binding.

### Hsa-miR-10a-5p overexpression governs resistance to translation stress

**Fig. 6:**
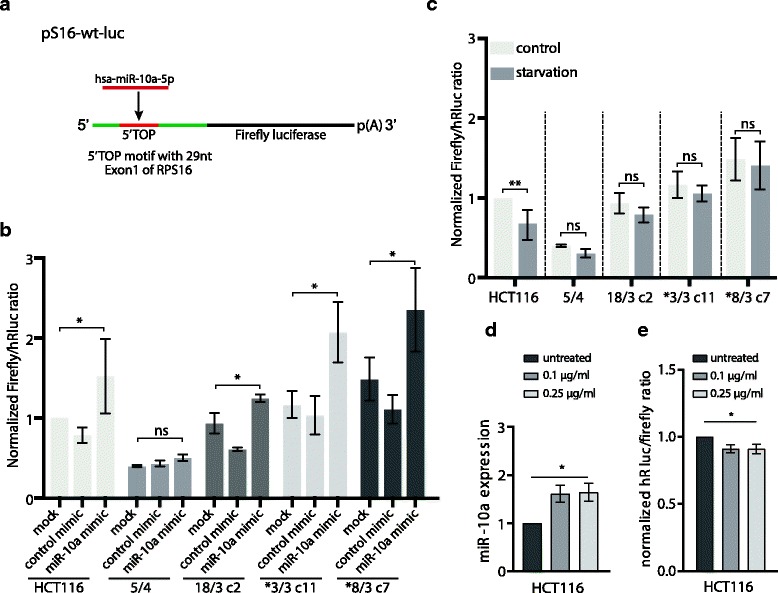
5’TOP motif reporter is less sensitive to starvation in aneuploid cells. **a** Schematic illustration of the pS-16-wt-luc luciferase reporter construct. RPS16 transcriptional start side and 29 nt of Exon 1 including the 5’TOP motif is incorporated into the 5’UTR of luciferase gene. **b** pS16-wt-luc activity after overexpression of hsa-miR-10a-5p. Cells were reverse transfected with hsa-miR-10a-5p mimic or control molecule and 24 h post transfection forward transfected with pS16-wt-luc and pRL-TK. 72 h post mimic transfection the luciferase assay was conducted. **c** pS-16-wt-luc activity after starvation. Cells were transfected as in B and serum- starved for 3 h before the luciferase reporter assay was conducted. Data presents Renilla normalized mean +/− SD values. Two-way ANOVA, multiple comparison correction with Sidak test. ns = not significant, *P < 0.0332, **P < 0.0021, ***P < 0.0002, ****P < 0.0001. **d** Abundance of has-miR-10a-5p determined by quantitative real-time PCR upon prolonged cyclohexamide treatment. **e** Endogenous hsa-miR-10a-5p activity upon prolonged cyclohexamide treatment determined by psiCheck2-10a luciferase reporter assay with hsa-miR-10a-5p binding site

Next, we considered the fact that hsa-miR-10a-5p binds also to the 5’UTR downstream of the 5’TOP motif that is found in mRNAs of ribosomal proteins (RP) and other mRNAs of translation associated proteins [19]. This binding results in enhanced translation of RP mRNAs and alleviates redistribution of RP mRNAs from active polysomes to inactive RNP complexes upon amino acid starvation. To analyse the translation efficiency of mRNAs with the 5’TOP motif, we employed a luciferase reporter containing the transcriptional start site of the ribosomal protein S16 (*Rps16*) and 29 nt of the exon 1 including the 5’TOP motif in the 5’UTR of a luciferase gene (pS16-wt-luc) (Fig. 6a, [19]). We first determined the steady state levels of 5’TOP motif mRNA translation with the pS16-wt-luc luciferase reporter and determined normalized Firefly luciferase signal similar to the wild type (Fig. 6b, mock control). Next, we measured the luciferase activity after overexpression of the hsa-miR-10a-5p mimic (Additional file 1: Figure S6A, B). Using the pS16-wt-luc luciferase reporter, we observed that hsa-miR-10a-5p overexpression enhanced the 5’UTR-mediated effect on the translation of 5’TOP motif mRNAs in the parental HCT116 cell line as well as in three out of four tested aneuploids (Fig. 6b). Thus, the expression of mRNAs with the 5’TOP motifs is sensitive to the changes in the hsa-miR-10a-5p abundance.

The translation of mRNAs with the 5’TOP motif is tightly regulated and cellular stresses such as starvation result in their translational repression and redistribution into inactive ribonucleoprotein complexes [40]. We hypothesized that upregulation of hsa-miR-10a-5p in aneuploid cell lines might protect the cells from the translational repression. To test this hypothesis, we measured the 5’TOP motif mRNA translation via the luciferase reporter assay in serum-deprived cells. Serum starvation led to a significant decrease of the luciferase activity in the parental HCT116 cell line. In contrast, all aneuploid cell lines were resistant to the translational reduction of the 5’TOP motif mRNAs (Fig. 6c). Aneuploidy is known to inhibit pathways related to ribosome and translation [12–15]. We found that even a short treatment with cycloheximide, a eukaryotic protein synthesis inhibitor, induced the expression of hsa-miR-10a-5p, whereas other stress conditions did not significantly affect its expression (Fig. 6d, e). Thus, we propose that the negative effect of aneuploidy on translation leads to induction of hsa-miR-10a-5p that may in turn facilitate survival of aneuploids by its positive effect on translation.

## Discussion

Using an integrated approach of large-scale miRNA, RNA and protein expression data analysis, we document deregulation of the miRNAome and its consequences in human aneuploid model cell lines (Fig. 1a). For the first time, we show that addition of one or two chromosomes results in extensive genome-wide miRNA expression changes in human cells (Fig. 1b-e). The data analysis reveals that the deregulated miRNAome inflicts negative effects on development, growth and proliferation, and may thereby contribute to the cellular response to aneuploidy. Moreover, our evidence suggests that upregulation of hsa-miR-10a-5p in aneuploid cell lines might present an adaptation to aneuploidy by providing means to selectively increase translation.

Karyotype copy number alterations were previously associated with deregulated miRNA expression in some cancers and miRNA coding regions show high frequency of copy number variations in ovarian, breast, melanoma and lung cancer [41, 42]. Interestingly, some studies report no correlation between the copy number status and miRNA expression in haematopoetic cancer and acute myeloid leukemia [43, 44]. We observed that the miRNA abundance does not readily increase according to the chromosomes copy number (Fig. 2, Additional file 1: Figure S2), which is in a contrast with mRNA expression changes that largely scale up with the chromosome copy number changes [12–15]. The lack of general correlation between miRNAs abundance and chromosome copy number changes suggests that the copy number changes are counteracted by a tight regulation of miRNA expression.

Aneuploidy remodels the cellular miRNAome, as 9 to 30% of miRNAs were differentially expressed in aneuploids compared to isogenic controls (Tables 1 and 2). Although the deregulated miRNAs are mostly unique to the individual aneuploid cell lines, the affected cellular pathways appear the same: analysis of the cellular functions affected by the deregulated miRNAs identified cellular development, growth and proliferation as common targets (Fig. 3a, b). The integrated data analysis showed that the majority of targets, which show an inverse expression to the deregulated miRNAs, are indeed downregulated on mRNA or protein level (Fig. 4, Additional file 1: Figure S3). Among the downregulated targets are proteins that are crucial for the cell cycle progression such as BUB1, CDC20, CDK4 and CHEK1 in HCT116 5/4 or Cyclin E1 and RB1 in RPE1-derived aneuploid cell lines. One of the targets, HMGA2, is strongly downregulated on both mRNA and protein level in HCT116 5/4 (Fig. 4b). HMGA2 is a validated target of hsa-miR-26a-5p, let-7a-5p and hsa-miR-125b-5p, all of which were found to affect cellular growth and proliferation. Downregulation of HMGA2 by hsa-miR-26a negatively affects cell proliferation and has tumour suppressive effects in gall bladder cancer [45]. Another strongly downregulated target is RCC2, which is targeted by hsa-miR-192-5p and hsa-miR-7-5p in HCT1165/4 and RPE1 5/3 12/3 (Fig. 4b, c). RCC2 is essential for mitotic spindle assembly and recently a novel function of RCC2 for G1-S transition has been recognized [46–48]. Aneuploidy impairs proliferation in most model systems [12, 13, 34] and this is reflected in the conserved transcriptional changes that include the downregulation of cell cycle associated pathways [14, 15]. Molecular mechanisms that lead to down-regulation of pro-proliferative genes and impaired proliferation in response to chromosome gain are poorly understood. Based on our results we propose that the miRNA deregulation contributes to the gene expression changes and cell cycle impairments observed in aneuploid cells.

Only a few miRNAs were commonly upregulated in response to aneuploidy, such as miR-139-5p or miR-10a-5p. Strikingly, many of these upregulated miRNAs are often downregulated in cancers. For example, hsa-miR-139-5p, a recognized tumor-suppressing miRNA, is downregulated in non-small cell lung cancer, gastric cancer, breast cancer and colorectal carcinoma and often associates with poor prognosis [39]. We found that inhibition of miR-139-5p decreases the expression of cell cycle inhibitors CDKN1A (p21) and CDKN1B (p27) (Fig. 3c). The opposite direction of expression changes in model aneuploids was previously observed for changes of mRNA levels, where the transcriptional response to chromosome gain is inversed to the cancer transcriptome [49]. Since gain of chromosomes has a potent tumor-suppressing effect [50], our findings suggest that the miRNAs deregulation contributes to this phenotype.

Among the few commonly upregulated miRNAs, we found hsa-miR-10a-5p and confirmed its increased endogenous activity using a specific luciferase-based reporter system (Fig. 6a, b). Only a few hsa-miR-10a-5p targets showed a concordant downregulation on protein or mRNA level in human aneuploid model cell lines. These few affected targets were involved in diverse cellular functions such as cytoskeleton stabilization, transcriptional regulation and metabolic processes (Additional file 1: Figure S5, Additional file 4: Table S3). This finding prompted us to investigate another possible function of hsa-miR-10a-5p. Hsa-miR-10a-5p binds upstream of the mRNA 5’TOP motif and affects the translation of mRNAs carrying this motif. The 5’TOP motif is a cis-regulatory motif present in transcripts encoding for the translational machinery, mostly ribosomal proteins [40, 51].

What is the function of the upregulated hsa-miR-10a-5p in aneuploid cells? Upon nutrition deprivation or cell cycle arrest the translation of 5’ TOP mRNAs is repressed, which is indicated by their shift to inactive ribonucleoprotein complexes [19]. We observed that the 5’TOP motif luciferase activity is not repressed upon starvation in aneuploid cells, in contrast to the diploid parental cell lines, where the 5’TOP motif luciferase activity is sensitive to starvation (Fig. 6c-e). As aneuploidy results in a plethora of cellular stresses, such as proteotoxic stress, metabolic and replication stress [52], we propose that the upregulation of hsa-miR-10a-5p in aneuploid cell lines might be a protective adaption to adverse stress conditions.

## Conclusions

Our unique data set describes the aneuploid cells from an “omics” perspective and allows a global, quantitative analysis of the consequences of aneuploidy in different cell lines. Our system demonstrates that an addition of extra chromosomes largely affects cell physiology on multiple levels, including marked deregulation of the miRNAome. The altered miRNAome landscape may explain some of the transcriptome and proteome changes that occur in response to aneuploidy and generally emphasizes the detrimental consequences of chromosome gains.

## Methods

### Cell lines

The retinal pigment epithelial cell line RPE1 hTERT and RPE1 hTERT H2B-GFP were a gift from Stefan Taylor (University of Manchester, UK). The human colorectal cell line HCT116 was obtained from American Type Culture Collection (no. CCL-247). HCT116 H2B-GFP was generated previously by lipofection (FugeneHD, Roche) transfection of pBOS-H2B-GFP (BD Pharmingen) according to manufacturer’s protocols [53]. The tetrasomic cell line HCT116 5/4 were a kind gift of Minoru Koi (Baylor University Medical Centre, Dallas, Texas, USA). All other trisomic and tetrasomic cell lines were generated by microcell-mediated chromosome transfer as described previously [5, 9, 12]. Cell lines originating from individual clonal populations arising from a single cell after by microcell-mediated chromosome transfer are indicated with their clone number (c#). The genome sequencing data of all cell lines are available in the public repository of NCBI under the accession number GSE102855. Summary of all cell line information is in Table 4.

**Table 4:** List of all cell lines used in the analysis

Cell line name	Origin	Full cell line name	Analysis	Remarks
HCT116	Koi laboratory		RNA and sRNA sequencing; qPCR	Kindly provided by Minoru Koi
HCT116 5/4	Koi laboratory		RNA and sRNA sequencing; qPCR	Kindly provided by Minoru Koi
HCT116 13/3 c2	MMTC into HCT116	HCT116 13/3 clone 2	qPCR	This work
HCT116 13/3 c3	MMTC into HCT116	HCT116 13/3 clone 3	qPCR	This work
HCT116 18/3 c1	MMTC into HCT116	HCT116 18/3 clone 1	qPCR	This work
HCT116 18/3 c2	MMTC into HCT116	HCT116 18/3 clone 2	sRNA sequencing; qPCR	This work
HCT116 21/3 c1	MMTC into HCT116	HCT116 21/3 clone 1	qPCR	This work
HCT116 21/3 c3	MMTC into HCT116	HCT116 21/3 clone 3	qPCR	This work
HCT116*	HCT116 from AATC introduction of H2B-GFP	HCT116 H2B-GFP	qPCR	(Kuffer et al., 2013) [53]
HCT116* 3/3 c11	MMTC into HCT116 H2B-GFP	HCT116 H2B-GFP 3/3 clone 11	sRNA sequencing; qPCR	(Passerini et al., 2016) [9]
HCT116* 3/3 c13	MMTC into HCT116 H2B-GFP	HCT116 H2B-GFP 3/3 clone 13	qPCR	(Passerini et al., 2016) [9]
HCT116* 5/3	MMTC into HCT116 H2B-GFP	HCT116 H2B-GFP 5/3	qPCR	(Stingele et al., 2012) [12]
HCT116* 8/3 c5	MMTC into HCT116 H2B-GFP	HCT116 H2B-GFP 8/3 clone 5	qPCR	(Donnelly et al., 2014) [5]
HCT116* 8/3 c6	MMTC into HCT116 H2B-GFP	HCT116 H2B-GFP 8/3 clone 6	qPCR	(Donnelly et al., 2014) [5]
HCT116* 8/3 c7	MMTC into HCT116 H2B-GFP	HCT116 H2B-GFP 8/3 clone 7	RNA and sRNA sequencing; qPCR	(Donnelly et al., 2014) [5]
HCT116* 8/3 c8	MMTC into HCT116 H2B-GFP	HCT116 H2B-GFP 8/3clone 8	qPCR	(Donnelly et al., 2014) [5]
RPE1	Taylor laboratory	RPE1 hTERT	RNA and sRNA sequencing; qPCR	Kindly provided by Steven Taylor
RPE1 5/3 12/3	MMTC into RPE1	RPE1 hTERT 5/3 12/3	RNA and sRNA sequencing; qPCR	(Stingele et al., 2012) [12] Spontaneous gain of chromosome 12
RPE1*	Taylor laboratory	RPE1 H2B-GFP hTERT	RNA and sRNA sequencing; qPCR	Kindly provided by Steven Taylor
RPE1* 21/3	MMTC into RPE1 H2B-GFP	RPE1 H2B-GFP hTERT 21/3	RNA and sRNA sequencing; qPCR	(Stingele et al., 2012 [12])
RPE1* 7/3	MMTC into RPE1 H2B-GFP	RPE1 H2B-GFP hTERT 7/3	sRNA sequencing; qPCR	This work Spontaneous gain of chromosomes 9,12
RPE1* 3/3	MMTC into RPE1 H2B-GFP	RPE1 H2B-GFP hTERT 3/3	sRNA sequencing; qPCR	This work

The asterisk in the cell line name marks the presence of H2B-GFP

### RNA sequencing and data processing

Following aneuploid cell lines and parental diploid cell lines were subjected to RNA sequencing: HCT116, HCT116 H2B-GFP (cell lines containing H2B-GFP will be indicated by * in the cell line name), HCT116 5/4, HCT116* 8/3 clone7, RPE1, RPE*, RPE1 5/3 12/3, RPE1* 21/3.

Total RNA was extracted using RNeasy Mini Kit (QIAGEN). TruSeq RNA library preparation and Illumina HiSeq2500 sequencing with 25 million 100 bp single reads per library were performed by the Max Planck-Genome-Center Cologne, Germany. Subsequently, sequencing adapters were removed from the raw sequences with cutadapt and sequencing reads were mapped to the human genome (hg19) using TopHat (v2.0.10) with the following parameters: “tophat2 -g1 -G”. RefSeq information in the GTF file was downloaded from the UCSC genome browser. featureCounts (v1.4.3) was used to generate the count matrix with the same GTF file as for the alignment with the following parameters: “-t exon -g gene_id”. Normalization and differential expression analysis was performed using the R/Bioconductor package DESeq2 [33]. For differential expression analysis, trisomic and tetrasomic cell lines were compared to the parental diploid cell line. The NGS data discussed in this publication have been deposited in NCBI’s Gene Expression Omnibus and are accessible through GEO Series accession number GSE102855 (https://www.ncbi.nlm.nih.gov/geo/query/acc.cgi?acc=GSE102855).”

### Small RNA sequencing and data processing

Following aneuploid cell lines and parental diploid cell lines were subjected to small RNA sequencing: HCT116, HCT116*, HCT116 5/4, HCT116 18/3 clone2, HCT116* 8/3 clone7, HCT116* 3/3, RPE1, RPE*, RPE1 5/3 12/3, RPE1* 21/3 and RPE1* 7/3.

Total RNA, including small RNAs from 18 nucleotides upwards, was extracted using miRNeasy Mini Kit (QIAGEN). TruSeq small RNA library preparation and Illumina HiSeq2500 sequencing with 25 million 100 bp or 75 bp single reads per library were performed by the Max Planck-Genome-Center Cologne, Germany. If necessary, raw sequencing read ends were trimmed for low quality base calls using the FASTQ quality trimmer with the following parameters: “-Q33 -t 20 -l 17” [28, 32]. Sequencing adapters were removed with cutadapt. Mapping to the human genome hg19 and miRNA identification was performed using mirdeep2 (v2.0.0.8) [32] with the following commands: “mapper.pl -e -h -i -j -l 18 -m -p hg19 -q” and “miRDeep2.pl reads.fa hg19.fa genome.arf human_mature_rmspace.fa others_mature_rmspace.fa human_hairpin_rmspace.fa -d -t Human”. The three biological replicates of aneuploid and the corresponding parental cell line were analyzed within the same analysis run. For subsequent normalization and differential expression analysis miRNA_expressed_all_samples.csv was used as an input for DESeq2. Differential expression analysis was performed between aneuploid cell lines and corresponding parental diploid cell line. To account for sequencing batch effects, paired replicate information in addition to condition information was input to the DESeq2 analysis for HCT116 5/4, RPE1 5/3 12/3 and RPE1* 21/3 and corresponding parental cell lines. The genome sequencing data of all cell lines are available in the public repository of NCBI under the accession number GSE102855.

### Integrative mRNA, miRNA and target analysis

Data analysis, such as integration and data visualization was performed using the computing environment R. Mapping of identifiers as well as genome locations was performed with BioMart using biomaRt package in R [54, 55]. miRNA (miRNA) identifiers were retrieved from miRBase (v21). miRNA target information was retrieved from miRTarBase (v6.1) [36, 56].

### Functional annotation analysis

miRNA differential expression datasets were analyzed using the Ingenuity Pathway Analysis Software [37]. Core analysis was performed with the differential expressed miRNAs (log2 fold change +/− 0.6, adjusted *p*-value < 0.05) against the Ingenuity knowledge base considering direct experimentally observed relationships in human species. Functional annotation analysis results were exported and visualized in R. Functional annotation analysis of target genes was conducted using the Database for Annotation, Visualization and Integrated Discovery (DAVID v6.8) by applying functional annotation clustering.

### Quantitative real-time PCR of miRNAs

Total RNA, including small RNAs from 18 nucleotides upwards, was extracted using miRNeasy Mini Kit (QIAGEN). Reverse transcription was performed using the universal cDNA synthesis kit miRCURY LNA™ (Exiqon) according to manufacturers protocol. UniSp6 RNA spike-in control was added to each sample in equal amounts. Quantitative PCR was conducted using the Light Cycler 480 System (Roche Diagnostics) using the ExiLENT SYBR® Green master mix miRCURY LNA™ (Exiqon) and miRNA specific LNA™ PCR primer sets (Exiqon) as well as the UniSp6 RNA spike control primer set (Exiqon). Absolute quantification with an external standard was performed and negative non-template controls were included in all experiments. The specificity of the primer product amplification was confirmed in each run by melting curve analysis. miRNA expression was normalized to the control spike RNA and corresponding diploid miRNA expression.

### Luciferase reporters

hsa-miR-10a-5p 3’UTR luciferase reporter (psiCheck2-10a) was constructed by cloning the complementary miRNA target sequence in the synthetic 3’UTR of psiCHECK™-2 Vector (Promega). PCR cloning was performed with the following primers:

top 5’-*CACAAATTCGGATCTACAGGGTAGTTTAAACCTAGAGCGGCCGCT*-‘3 and.

bottom 5’-*CTGACCTATGAATTGACAGCCGCGATCGCCTAGAATTACTGC*-‘3.

pS16-WT luciferase vector containing the transcriptional start site of the ribosomal protein S16 (Rps16) and 29 nt of the exon 1 including the 5’TOP motif was a kind gift of Anders H. Lund (University of Copenhagen, Denmark). pRL-TK Renilla vector was a kind gift of Reinhard Fässler (Max Planck Institute of Biochemistry, Martinsried).

### Endogenous hsa-miR-10a-5p mediated 3’UTR repression assay

Cell lines were forward transfected with psiCheck2-10a using Lipofectamine 200 (Thermo Fisher Scientific) according to manufacturer’s protocol. Cells were reseeded into 96 well plates 24 h (hrs) post transfection at 20.000/ well HCT116- derived cell lines and 10.000/ well RPE1 derived cell lines. Luciferase activity was monitored 48 h post transfection using the Dual-Glo® Luciferase Assay System (Promega). Renilla luciferase activity values were normalized to the Firefly luciferase activity and subsequently to the parental cell line. Statistical testing and data plotting was performed in GraphPad Prism 6.

### Hsa-miR-10a-5p mediated 5’TOP motif mRNA translation assay

Cell lines were reverse transfected with miRCURY LNA™ miRNA Mimic or miRCURY LNA™ miRNA Mimic Negative Control (Exiqon) at 50 nM using Lipofectamine 200 (Thermo Fisher Scientific) according to manufacturers protocol. Cells were forward transfected with the pS16-wt-luc Firefly luciferase reporter construct and pRL-TK Renilla luciferase control vector 24 h post mimic transfection. Forty-eight hours post mimic transfection, cells were reseeded into 96 well plates at 20.000/ well HCT116- derived cell lines and 10.000/ well RPE1- derived cell lines. Luciferase activity was monitored 72 h post transfection using Dual-Glo® Luciferase Assay System (Promega). Starvation was performed by replacing the Dulbecco’s Modified Eagle Medium (DMEM) supplemented with 10% fetal bovine serum and 1% penicillin/ streptomycin with DMEM without supplements for 3 h prior measurement of luciferase activity. Firefly luciferase activity values were normalized to Renilla luciferase activity and subsequently to the parental cell line. Statistical testing and data plotting was performed in GraphPad Prism 6.

## Additional files

Additional file 1: **Figure S1.** Correlation of extra DNA and the percentage of deregulated microRNAs for each aneuploid cell line. **Figure S2.** microRNA expression aligned according to their chromosome position. Summary of all microRNA expression in analyzed aneuploid cell lines. **Figure S3.** microRNAs and their targets deregulated in aneuploids. Graphic presentation of the miRNAs deregulation relative to their targets in aneuploid human cells. **Figure S4.** Comparison of hsa-miR-10a-5p expression and HOXB3 mRNA expression. The correlation between expression of hsa-miR-10a-5p and its hosting genomic sequence. **Figure S5.** Target mRNA and protein expression levels. Graphic presentation of the hsa-miR-10a deregulation relative to its targets in aneuploid human cells. **Figure S6.** Overexpression of hsa-miR-10a-5p leads to repression of luciferase activity. Validation of the hsa-miR-10a-5p overexpression in human cells. (PDF 250 kb)

Additional file 2: **Table S1.** Total number and number of deregulated microRNAs per chromosome (log2 fold change > 0.6 or <(− 0.6)). Overview of all deregulated microRNAs. (DOCX 16 kb)

Additional file 3: **Table S2.** Functional annotation clustering. Complete results of the DAVID analysis of functional annotation clustering of microRNAs targets deregulation. (XLSX 114 kb)

Additional file 4: **Table S3.** Functional annotation clustering of hsa-miR-10a-5p targets. Complete results of the DAVID analysis of functional annotation clustering of hsa-miR-10a-5p target deregulation. (XLSX 47 kb)

## Abbreviations

3’UTR: 3′ untranslated region
5’UTR: 5′ untranslated region
IPA: Ingenuity pathway analysis
miRNA: micro ribonucleic acid
PCR: Polymerase chain reaction
RP: Ribosomal proteins
