# Supplementary material for: The deregulated microRNAome contributes to the cellular response to aneuploidy
Source: BMC Genomics. 2018 Mar 14;19:197. doi: 10.1186/s12864-018-4556-6 (PMC6389165; doi:10.1186/s12864-018-4556-6)
Supplement: Supplementary file 1 — Figure S1. Correlation of extra DNA and the percentage of deregulated microRNAs for each aneuploid cell line. Figure S2. microRNA expression aligned according to their chromosome position. Summary of all microRNA expression in analyzed aneuploid cell lines. Figure S3. microRNAs and their targets deregulated in aneuploids. Graphic presentation of the miRNAs deregulation relative to their targets in aneuploid human cells. Figure S4. Comparison of hsa-miR-10a-5p expression and HOXB3 mRNA expression. The correlation between expression of hsa-miR-10a-5p and its hosting genomic sequence. Figure S5. Target mRNA and protein expression levels. Graphic presentation of the hsa-miR-10a deregulation relative to its targets in aneuploid human cells. Figure S6. Overexpression of hsa-miR-10a-5p leads to repression of luciferase activity. Validation of the hsa-miR-10a-5p overexpression in human cells. (PDF 250 kb) [file 12864_2018_4556_MOESM1_ESM.pdf]

## Supplementary Figures

### Supplementary Figure 1

Correlation of extra DNA and the percentage of deregulated microRNAs for each aneuploid cell line.

### Supplementary Figure 2

microRNA expression aligned according to their chromosome position

### Supplementary Figure 3

microRNAs and their targets deregulated in aneuploids

### Supplementary Figure 4

Comparison of hsa-miR-10a-5p expression and HOXB3 mRNA expression.

### Supplementary Figure 5

hsa-miR-10a targets mRNA and protein expression levels.

### Supplementary Figure 6

Overexpression of hsa-miR-10a-5p leads to repression of luciferase activity

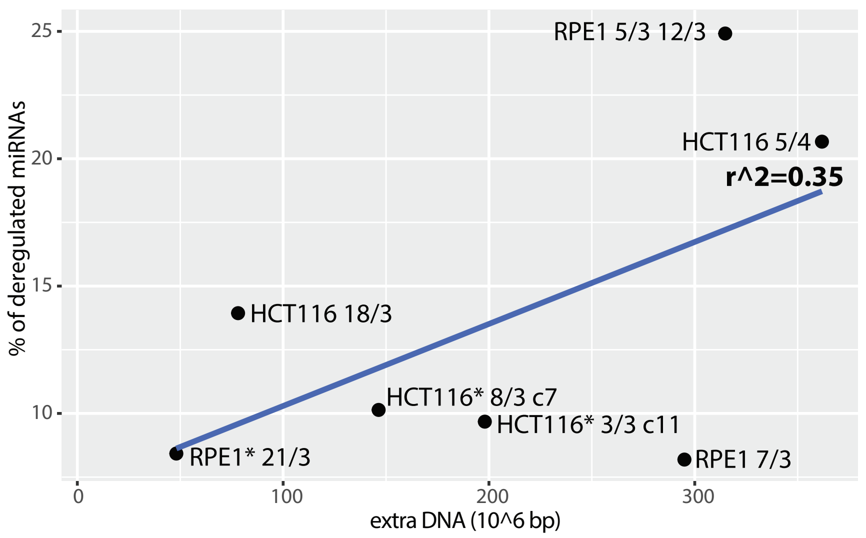


**Supplementary Figure 1**. Correlation of the amount extra DNA and the percentage of deregulated microRNAs in aneuploid cell lines. Extra DNA was estimated as the bp length of the aneuploid chromosome. Blue line presents fitted linear regression with R squared=0.35.

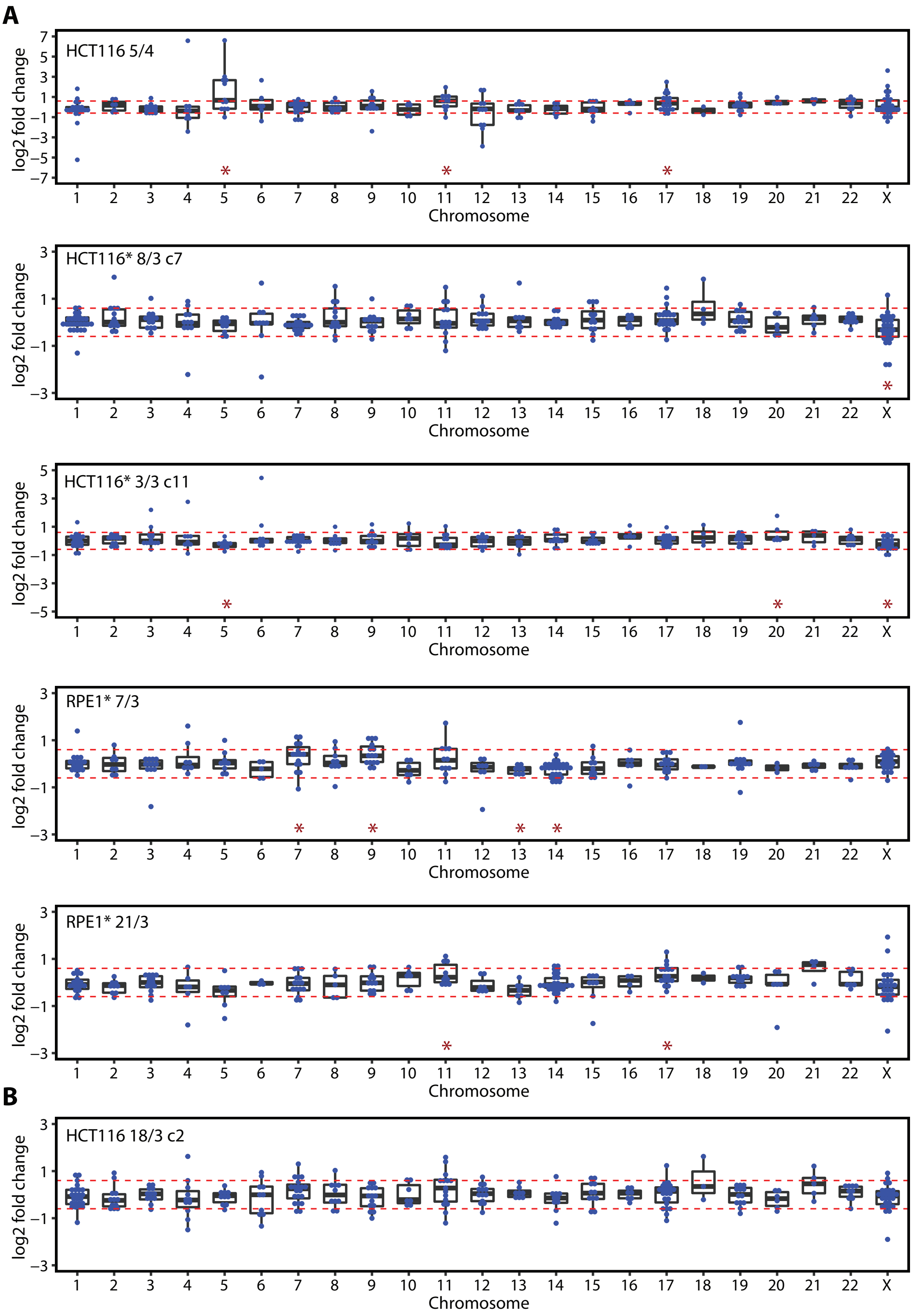


**Supplementary Figure 2.** microRNAs expression aligned according to their chromosome position. **A** HCT116 5/4, HCT116* 8/3 c7, HCT116* 3/3 c11, RPE1* 7/3 and RPE1* 21/3 with significantly increased or decreased median expression of chromosomes labelled with a red asterisks (Mann-Whitney-Wilcoxon test p-value <0.05). **B** HCT116 18/3 c2 does not show altered median miRNA expression of any chromosomes. Asterisks in the name of the cell line indicate cell lines expressing H2B-GFP. Each blue dot represents a microRNA with its chromosome location (x-axis) and log2 fold change normalized to corresponding parental cell line (y-axis). Red lines indicate log2 fold change >0.6/ <-0.6.
Boxplot present 75%, 50% and 25% quantile of the microRNA expression per chromosome.

Durrbaum et al
Supplementary Figure 2

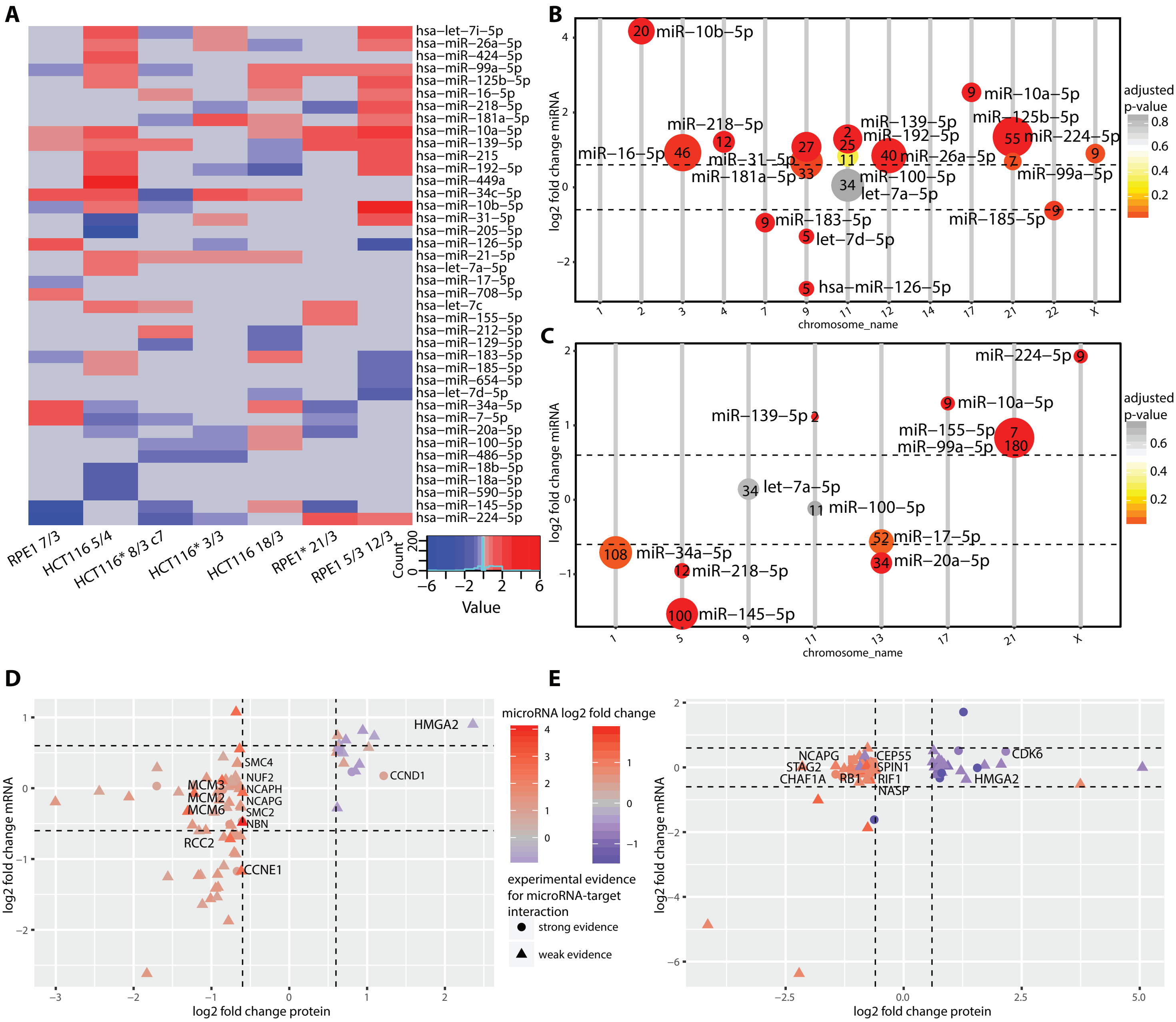


**Supplementary Figure 3. A** Heatmap of microRNAs associated with molecular function term "Cellular Development, Cellular Growth and Proliferation". Blue indicates downregulation, red upregulation. **B** Number of targets with strong evidence for each deregulated microRNA annotated in the "Cellular Development, Cellular Growth and Proliferation" category in RPE1 5/3 12/3. The size indicates the number of targets, the colour shows the significance of microRNA deregulation. The microRNAs are presented according to their chromosome location. **C** same as B in RPE1* 21/3. **D** Target genes within the "Cellular Development, Cellular Growth and Proliferation" category and their mRNA and protein expression. Selected gene labels indicate targets with inverse micRNA-target expression which are associated with cell cycle processes. Shape indicates the type of experimental evidence for a microRNA-target interaction. The colour indicates log2 fold change microRNA expression in RPE1 5/3 12/3 and **E** in RPE1 H2B-GFP 21/3.

Durrbaum et al
Supplementary Figure 3

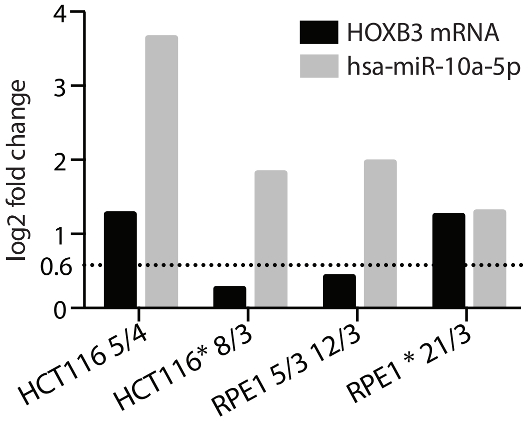


**Supplementary Figure 4.** Comparison of hsa-miR-10a-5p expression and HOXB3 mRNA expression. Bars represent log2 fold change expression ratio (aneuploid/ parental cell line) derived from RNA and sRNA sequencing.

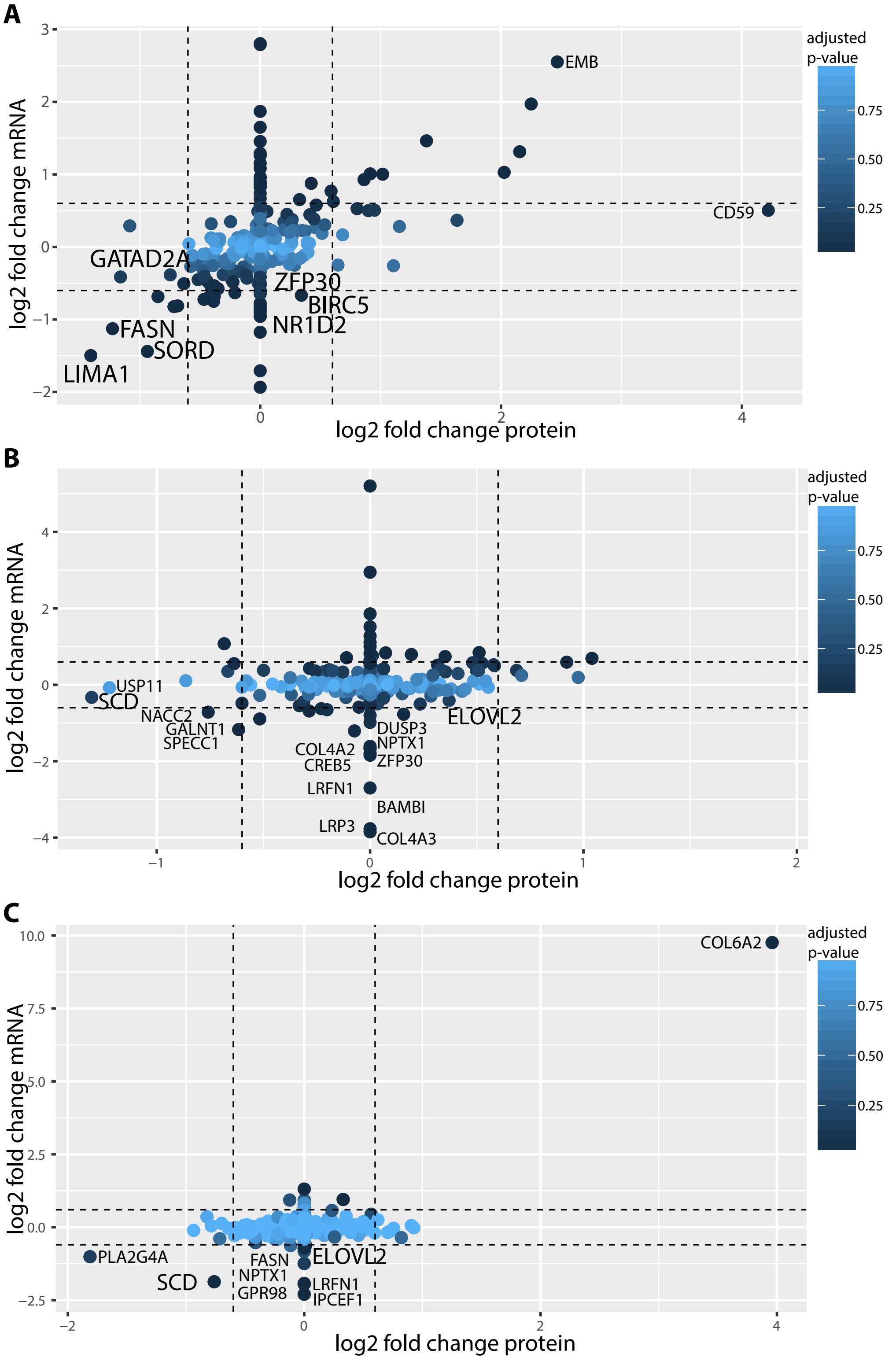


**Supplementary Figure 5.** hsa-miR-10a targets mRNA and protein expression levels. **A** hsa-miR-10a-5p target expression in HCT116 5/4 on protein level (x-axis) and mRNA level (y-axis). Each dot represents one target, color indicates the adjusted p-value for the mRNA levels.Dotted lines indicate log2 fold change of 0.6 and -0.6. **B** hsa-miR-10a-5p target expression in RPE1 5/3 12/3. **C** hsa-miR-10a-5p target expression in RPE1* 21/3. Asterisks indicate H2B-GFP.

Durrbaum et al
Supplementary Figure 5

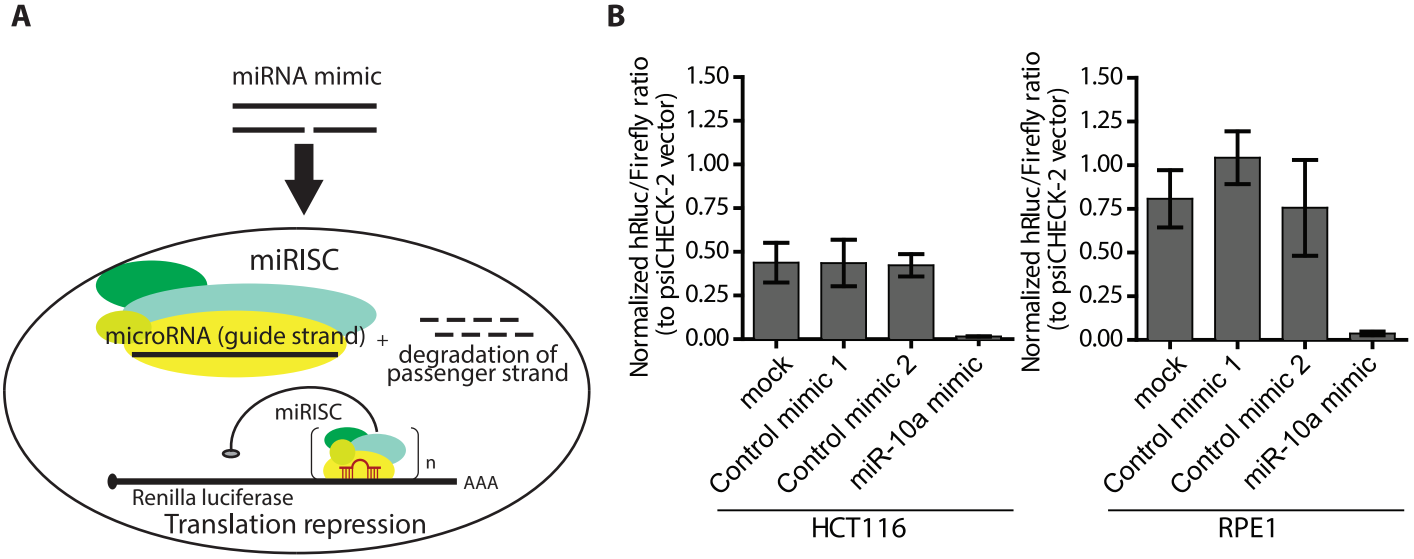


**Supplementary Figure 6**. Overexpression of hsa-miR-10a-5p leads to repression of luciferase activity. **A** Schematic illustration of the mechanism of overexpression via miRNA mimic molecule transfection. Mimic transfection leads to enhanced translational repression of target molecules by incorporation of the guide strand into the miRISC. **B** hsa-miR-10a-5p mimic transfection results in repression of Renilla luciferase activity in HCT116 and RPE1 cell lines. Cell lines were transfected with hsa-miR-10a-5p and control mimic molecules.  24 hrs post transfection, cell lines were forward transfected with psiCheck2 luciferase reporter construct. Luciferase reporter assay was conducted 72 hrs post mimic transfection. Data present Firefly luc normalized mean values +/- SD.

Durrbaum et al
Supplementary Figure 6
