# Supplementary material for: The deregulated microRNAome contributes to the cellular response to aneuploidy
Source: BMC Genomics. 2018 Mar 14;19:197. doi: 10.1186/s12864-018-4556-6 (PMC6389165; doi:10.1186/s12864-018-4556-6)
Supplement: Supplementary file 2 — Table S1. Total number and number of deregulated microRNAs per chromosome (log2 fold change > 0.6 or <(− 0.6)). Overview of all deregulated microRNAs. (DOCX 16 kb) [file 12864_2018_4556_MOESM2_ESM.docx]

**Supplementary Table 1** Total number and number of deregulated microRNAs per chromosome (log2 fold change >0.6 or <(-0.6)

| **Chromosomes** | | **1** | **2** | **3** | **4** | **5** | **6** | **7** | **8** | **9** | **10** | **11** | **12** | **13** | **14** | **15** | **16** | **17** | **18** | **19** | **20** | **21** | **22** | **X** |
| --- | --- | --- | --- | --- | --- | --- | --- | --- | --- | --- | --- | --- | --- | --- | --- | --- | --- | --- | --- | --- | --- | --- | --- | --- |
| **HCT116 5/4** | Total microRNAs | 20 | 7 | 14 | 9 | 12 | 6 | 20 | 11 | 11 | 6 | 11 | 11 | 9 | 7 | 9 | 5 | 20 | 2 | 13 | 4 | 3 | 9 | 29 |
|  | Deregulated microRNAs | 5 | 3 | 2 | 5 | 8 | 3 | 6 | 1 | 4 | 3 | 7 | 6 | 2 | 3 | 4 | 2 | 9 | 1 | 3 | 1 | 2 | 5 | 13 |
| **HCT116 18/3 c2** | Total microRNAs | 24 | 12 | 12 | 11 | 10 | 10 | 20 | 10 | 14 | 7 | 15 | 13 | 8 | 10 | 11 | 7 | 22 | 3 | 13 | 6 | 5 | 10 | 27 |
|  | Deregulated microRNAs | 4 | 4 | 1 | 4 | 1 | 6 | 6 | 3 | 3 | 1 | 8 | 2 |  | 3 | 4 |  | 4 | 1 | 1 | 1 | 2 |  | 5 |
| **HCT116* 3/3 c11** | Total microRNAs | 21 | 13 | 14 | 9 | 11 | 11 | 20 | 11 | 13 | 7 | 13 | 12 | 9 | 13 | 11 | 7 | 20 | 4 | 13 | 6 | 5 | 9 | 26 |
|  | Deregulated microRNAs | 3 | 3 | 2 | 1 | 2 | 1 | 2 | 2 | 2 | 1 | 1 | 2 | 2 |  | 1 | 1 | 1 | 1 | 2 | 2 | 1 | 3 |  |
| **HCT116* 8/3 c7** | Total microRNAs | 27 | 13 | 13 | 11 | 10 | 9 | 19 | 13 | 14 | 7 | 14 | 14 | 8 | 12 | 12 | 7 | 22 | 4 | 14 | 6 | 6 | 10 | 26 |
|  | Deregulated microRNAs | 2 | 3 | 1 | 3 | 1 | 2 |  | 4 | 2 | 2 | 5 | 2 | 1 |  | 4 |  | 4 | 1 | 1 | 1 | 1 |  | 8 |
| **RPE1 5/3 12/3** | Total microRNAs | 14 | 9 | 14 | 6 | 12 | 5 | 14 | 6 | 13 | 6 | 13 | 9 | 7 | 27 | 6 | 5 | 17 | 2 | 11 | 5 | 4 | 6 | 26 |
|  | Deregulated microRNAs | 8 | 3 | 5 | 3 | 9 | 2 | 2 | 2 | 8 | 3 | 5 | 6 | 1 | 10 | 3 | 2 | 11 | 3 | 1 | 2 | 5 | 13 |  |
| **RPE1* 7/3** | Total microRNAs | 17 | 8 | 12 | 8 | 10 | 5 | 16 | 9 | 15 | 6 | 11 | 8 | 8 | 29 | 9 | 6 | 14 | 2 | 13 | 3 | 6 | 8 | 24 |
|  | Deregulated microRNAs | 1 | 1 | 1 | 2 | 2 | 1 | 6 | 3 | 5 | 1 | 4 | 1 |  | 4 | 2 | 1 | 1 |  | 2 |  |  | 1 | 4 |
| **RPE1* 21/3** | Total microRNAs | 18 | 9 | 14 | 6 | 8 | 3 | 15 | 5 | 12 | 6 | 10 | 7 | 7 | 28 | 8 | 4 | 16 | 2 | 12 | 6 | 4 | 7 | 24 |
|  | Deregulated microRNAs | 1 | 1 | 1 | 2 | 2 |  | 4 | 2 | 2 | 1 | 3 |  | 1 | 3 | 1 |  | 5 |  | 2 | 1 | 3 |  | 6 |
